# Reliability-aware ensemble recommendation for open-network commerce: a dual-metric framework for jointly evaluating ranking accuracy and serving correctness

**DOI:** 10.3389/fdata.2026.1911540

**Published:** 2026-10-08

**Authors:** Rish Praveen S, Vani Rajasekar

**Affiliations:** School of Computer Science and Engineering, Vellore Institute of Technology (VIT), Vellore, Tamil Nadu, India

**Keywords:** Beckn protocol, dual-metric evaluation, ensemble learning, fallback mechanisms, graph collaborative filtering, neural collaborative filtering, Open Network for Digital Commerce (ONDC), recommender systems

## Abstract

Recommendation quality in open-network commerce is conventionally measured through offline ranking metrics that implicitly assume reliable upstream retrieval, stable item identities, and consistent catalog availability. In open networks such as India's Open Network for Digital Commerce (ONDC), these assumptions are routinely violated: catalogs are contributed by heterogeneous, independently operated providers, transactions follow asynchronous Beckn-style protocol lifecycles, and identifiers drift across service boundaries. This paper presents LocalMarket, a reliability-aware recommendation platform (Next.js frontend, FastAPI backend, ensemble ranking layer) that implements ONDC-pattern transaction flows in a controlled, repository-reproducible environment. We propose and validate a dual-metric evaluation framework that decouples model effectiveness—measured through Mean Average Precision (MAP@10), Recall@10 and Normalized Discounted Cumulative Gain (NDCG@10)—from serving correctness, measured through a novel Route Resolution Success Rate (RRSR) and Wishlist Consistency Rate (WCR). Using a controlled within-system ablation across Model-only, Reliability-only, and Combined configurations on fixed traffic traces, we utilize a convex-combination ensemble containing neural collaborative filtering (NCF) (utilizing only GMF embeddings for retrieval latency), graph-based, session-aware, and content-based components primarily as a vehicle for testing this framework. While this ensemble configuration improves NDCG@10 by 70.7% over a Popularity-based baseline, our primary finding validates that four targeted reliability interventions raise RRSR from 60.0 to 99.8% and WCR from 0.0 to 100.0%. Critically, the combined configuration preserves both gains simultaneously, indicating that model-level and serving-level improvements are additive rather than substitutive. The results support a systems-level thesis: in open-network commerce recommenders, ranking accuracy and serving correctness are independent, jointly necessary axes of quality, and reporting only one materially overstates the value delivered to end users. We further contribute a reusable integration failure-mode taxonomy, a constrained ensemble-weight selection protocol, and a reproducible experiment-replay procedure intended to generalize to other open-network commerce deployments.

## 1 Introduction

### 1.1 Background

A recommendation system shapes what users see, what they consider, and ultimately what they buy. Because of this gate-keeping role, the reliability of a recommender—its ability to consistently surface usable, navigable, and state-consistent results—is arguably as important as the relevance of the items it ranks. Yet the dominant evaluation paradigm in the recommender-systems literature focuses almost exclusively on offline ranking quality, computed under the implicit assumption of a fixed data contract, stable item identities, and uninterrupted access to upstream catalog data (He et al., 2017, 2020; Jannach and Ludewig, 2020). These assumptions are reasonable for closed, single-operator platforms where the recommender, the catalog service, and the storefront are developed and deployed by the same organization.

Open-network commerce breaks this assumption set. Initiatives such as India's Open Network for Digital Commerce (ONDC) and the underlying Beckn protocol define an architecture in which buyer applications, seller applications, logistics providers, and technology service providers interoperate as independent network participants connected through standardized but asynchronous protocol messages (Beckn Protocol Foundation, 2022a,b; ONDC Official, 2022a,b). In such an ecosystem, the catalog seen by a recommender is an aggregation across many independently operated providers, each with its own schema conventions, availability characteristics, and identifier spaces. A request for product details, a route to a product page, or a wishlist toggle may traverse several of these boundaries before it reaches the user, and each boundary is a potential point of failure that is entirely orthogonal to the quality of the underlying ranking model.

This paper introduces LocalMarket, a reliability-aware recommendation platform designed explicitly for this open-network scenario. LocalMarket combines a Next.js frontend, a FastAPI backend, and an ensemble model layer that fuses neural collaborative filtering (NCF), graph-based collaborative filtering, session-aware sequence modeling, and content-based priors. The platform implements ONDC-pattern (Beckn-style) transaction flows—search, select, init, and confirm—in a controlled local environment, and treats robust service delivery as an intrinsic dimension of recommendation quality rather than an operational afterthought (Beckn Protocol Foundation, 2022a,b; ONDC Official, 2022a).

### 1.2 Problem context

Independent variation among catalog providers, buyer applications, and recommendation services in an open network gives rise to failure modes that are rarely represented in benchmark recommendation datasets. We identify four recurring classes of such failures:

- Provider heterogeneity and cross-domain schema mismatches, where different sellers expose product payloads with inconsistent field names, types, or units.
- Sporadic upstream unavailability, throttling, and asynchronous result delivery, which can leave a recommendation pipeline waiting on, or silently dropping, data from a slow or unreachable provider.
- Cross-service and cross-client identifier heterogeneity, in which the same logical product is referenced by different identifier types (numeric vs. string) or different identifier bands across the frontend, backend, and external catalog.
- Catalog volatility and inconsistent state, which destabilizes the deterministic behavior that many ranking models implicitly assume during both training and inference.

Now, to make this clear and formal, let P = {p_1_,…,p_m_} be the independent network participants (catalog providers), each p_j_ revealing a locally-defined catalog C_j_ with a provider-local schema σ_j_, and a provider-local identifier space I_j_. But since the platform catalog is a piecewise-gathered construction, in that it is not hand-crafted and maintained by a single participant (or system operator), this catalog will be well-formed provided that for every item there are two additional maps which succed: a schema-normalizing map ψ which brings each provider-local payload into the platform schema and an identifier-resolution map ϕ which resolves provider-local identifiers into a single global identifier space. We write this formally in Equations C1, C2, and C3:


$$\begin{array}{l} \hat{C}\;=\;\psi \left(\sigma_{1},\;C_{1}\right)\;\cup \;\psi \left(\sigma_{2},\;C_{2}\right)\;\cup \ldots \cup \;\psi \left(\sigma_{m},\;C_{m}\right) \end{array}$$ (C1)

$$\begin{array}{l} \varphi :\;I_{j}\;\to \;I_{global},\;j\;=\;1,\ldots ,\;m \end{array}$$ (C2)

$$\begin{array}{l} r_{k}\;=\;s\left(i_{k}\right),\;1\left[r_{k}\;ok\right]\;=\;1\;if\;s\left(i_{k}\right)\text{ resolves to a valid},\\ \text{ displayable page},\text{ else }0 \end{array}$$ (C3)

A request of the user u for recommendations leads to a ranked list L(u) = (i_1_,…,i_K_) ranked with respect to the normalized catalog C; a serving function s is needed to send L(u) to the user by transforming the represented identifiers in each i_k_ to navigable routes r_k_ on the catalog state when C is resolved, possibly different than it was when ranking. This makes explicit the indicator in C3 for a particular item: 1[r_k_ ok]. Each of the four failure classes listed above occurs at one of four distinct places that the entire pipeline can break: ψ is undefined for the given payload, class i; cannot reach provider p_j_ when C_j_ is queried, class ii; ϕ fails to map two representations of the identical conceptual item to one global ID, class iii; Catalog-volatility/serving: C differs from when to when C is resolved, class iv.

Basic ranking metrics are purely functions of L(u), 1[r_k_ ok] = 1 for all K, but, since RRSR and WCR (defined formally as Section 4.3.2) are functions of 1[r_k_ ok] directly—or the consistency ϕ gives the identifier—they can “see” what basic ranking cannot as inherent limitations of the pipeline.

Figure 1 graphs the pipeline and where [as B1 (provider/schema), B2 (identifier), or B3 (catalog-volatility/serving)] and what kind of failure occurs.

**Figure 1:**
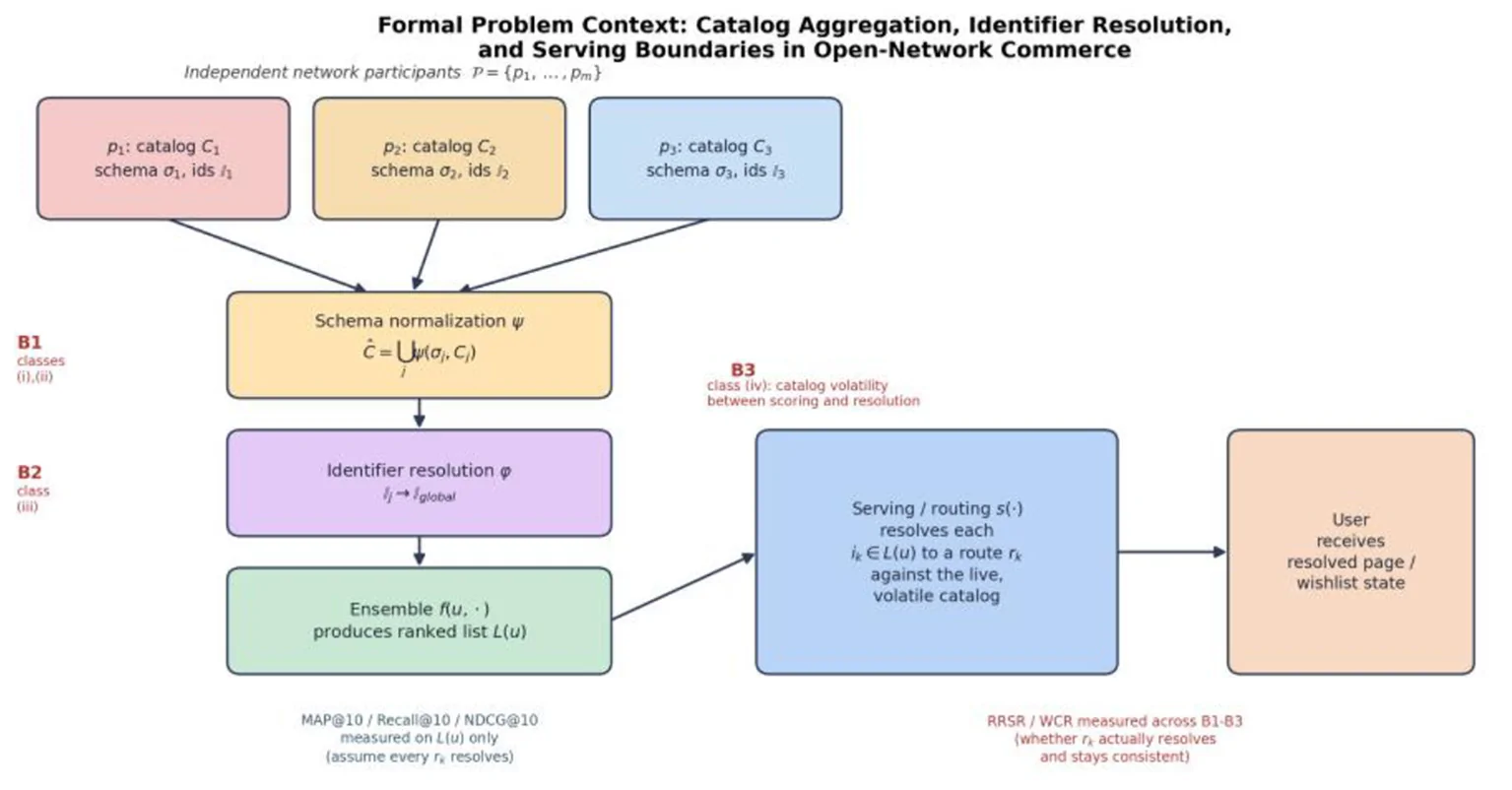
Formal problem context: independent providers p_1_,….,p_m_ are aggregated through schema normalization ψ and identifier resolution ϕ before being scored by the ensemble; serving/routing then resolves the scored list against the live catalog. Boundaries B1–B3 mark where failure classes (i)–(iv) originate; ranking metrics are computed on L(u) alone, while RRSR and WCR are computed across B1–B3.

When any of these failure modes reaches the user interface, the overall quality of the recommendation experience deteriorates regardless of the ranking score that produced it. A top-ranked item is of no value to a user if its detail page cannot be resolved, if the route to that page is broken, or if the item's state is inconsistent between the cart and wishlist views (Jannach and Ludewig, 2020; Herlocker et al., 2004). Beckn- and ONDC-style protocol roles and asynchronous lifecycles widen the integration surface across which such failures can occur (Beckn Protocol Foundation, 2022a,b; ONDC Official, 2022a), making this an especially acute concern for open-network commerce recommenders.

Existing evaluation practice does not adequately capture this. Standard offline ranking metrics—Hit Rate (HR@K), NDCG@K, Recall@K, and Mean Reciprocal Rank (MRR@K)—quantify the quality of an ordering assuming that the ordered items are retrievable, displayable, and actionable. None of these metrics is sensitive to a broken product route, a fallback that silently truncates the candidate set, or a wishlist toggle that is lost because of an identifier-type mismatch. This thesis therefore evaluates recommendation quality along two explicitly decoupled axes:

- Model quality—the ranking-centric performance offered by hybrid recommendation models, measured with MAP@10, Recall@10 and NDCG@10.
- Serving quality—the user-facing correctness of recommendations in the presence of failures and fallbacks, measured with RRSR and WCR.

### 1.3 Research questions

This work addresses three research questions (RQs):

- **RQ1**. Does an ensemble model outperform individual base models from the same repository in terms of offline ranking quality under a static protocol?
- **RQ2**. Are targeted reliability interventions independently effective at improving user-visible serving correctness, relative to a non-intervention baseline?
- **RQ3**. When model-level and reliability-level interventions are combined within the same system, do their effects add, cancel, or render one another redundant in a controlled experiment?

### 1.4 Motivation and contributions

The motivation for this work rests on four concerns that are common to production recommender deployments but under-represented in academic evaluation: (i) product continuity—users should receive a coherent shopping experience irrespective of the health of a fragmented, dynamic catalog; (ii) engineering quality—routing errors, identifier mismatches, and other defects that arise at integration boundaries with external services must be treated as first-class quality issues; (iii) scientific rigor—the marginal contribution of a better model should be distinguishable, on a fixed benchmark, from the marginal contribution of a more reliable serving layer; and (iv) deployment relevance—the resulting artifact and protocol should be directly reproducible from a public code repository.

It should be highlighted that the ensemble is not one where the four standard families of models are simply united for unity's sake. Instead, each part is present for the simple reason that it's the part of a model most appropriately suited to one of the four ONDC-specific failures defined in Section 1.2, and removing it reopens our vulnerability to one such failure class. The GMF branch of NCF is kept, for it learns only on the identities in interactions and requires no shared product—attribute schema-meaning it's resilient to the very provider heterogeneity and schema mismatches of failure class (i) that limit effectiveness in attribute-only methods over independently operated shops.

The lightGCN is kept, for higher order graph propagation recover signal for sparsely encountered items and complement sporadically lost upstream service signals [failure class (ii)] that would otherwise shorten, unseen, the logged interactions that a first-order model would exploit.

GRU4rec is kept, for the reliance on a local session and not a global or cross-service user state makes it the component least susceptible to cross-service identifier drift [failure class (iii)]-since a session's identifier may remain stable even as product representation shifts. The content-based component is kept for it's ability to score an item at the instant it enters existence, prior to any collaborative signal being available, which are just the circumstances occasioned by catalog volatility and vendor churn, [failure class (iv)] for which we need such resilience. Four component branches align naturally with the four bug classes defined in Table 1, and so the 4 layers of the Architecture described in Table 2 and the 4 failure classes described in Section 1.2-they are mapped 1 to 1 for testability; thus, we view our contributions to be the construction of this mapping, and its testing under the dual-metric, rather than the individual merit of any model families.

**Table 1:** Bug-class remediation summary with measurable effect focus.

Failure-mode class	System symptom	Technical mitigation	Impacted metric
Degradation-window failure	Candidate collapse in fallback mode	Limit-aware fallback generation	Candidate adequacy
Contract-resolution failure	Wrong route for high-band item IDs	Full ID-band category mapping	RRSR
Schema-type-boundary failure	Wishlist state mismatch across identifier types	Boundary-level identifier normalization	WCR
Invocation-contract failure	Incorrect similar-item utility call	Corrected invocation contract	Similar-item success rate

**Table 2:** Architecture layer responsibilities and quality objectives.

Layer	Primary responsibilities	Quality objective
Presentation	Product discovery UI, recommendation rendering, transaction interactions	Usability and continuity
Frontend data	API adapters, fallback orchestration, client-side identifier normalization	Route correctness
Service layer	Recommendation APIs, catalog APIs, ONDC lifecycle, review endpoints	Availability and contract stability
Model/data layer	Ensemble scoring, candidate retrieval, caching and persistence	Ranking quality and latency

In light of these concerns, this paper makes the following contributions:

- **A dual-metric evaluation framework** that decouples ranking effectiveness from serving correctness, together with formal definitions of RRSR and WCR.
- **A within-system, controlled-ablation protocol** comparing Model-only, Reliability-only, and Combined configurations on fixed traffic traces. A convex-combination ensemble—including an NCF component utilizing only GMF embeddings to maintain retrieval latency—is utilized as the vehicle for testing this framework (Sections 4 and 6).
- **A reusable taxonomy** of four integration failure-mode classes—degradation-window, contract-resolution, schema-type-boundary, and invocation-contract failures—together with their corresponding engineering mitigations (Section 5).
- **A reliability-aware reference architecture** for recommendation in open-network commerce settings, comprising a layered presentation, frontend-data, service, and model/data architecture with an explicit three-tier fallback hierarchy (Section 3).
- An artifact-level, repository-reproducible replay protocol that allows the reported experiments to be regenerated end-to-end (Section 6). The full source code, data preprocessing scripts, and deployment configurations are publicly available under the MIT License at: https://github.com/Rishpraveen/localMarket_beckn.

### 1.5 Research significance and paper organization

This work extends recommendation-system evaluation from ranking-only reporting toward a model-plus-serving perspective that is aligned with the operational realities of open-network commerce (Beckn Protocol Foundation, 2022a; Press Information Bureau, 2024, 2025). It further provides a reproducible methodology for separating model effects from serving effects through controlled ablation—a practice that remains underreported in conventional recommender evaluation (He et al., 2017; Jannach and Ludewig, 2020). The remainder of the paper is organized as follows. Section 2 reviews related work in neural, graph-based, session-based, and hybrid recommendation, as well as reliability engineering for distributed systems. Section 3 presents the LocalMarket architecture, the ensemble scoring formulation, and the reliability mechanisms. Section 4 describes the dual-metric evaluation methodology, including formal metric definitions and the experimental protocol. Section 5 details the implementation. Section 6 reports and discusses the experimental results. Section 7 discusses threats to validity, Section 8 addresses ethics and data governance, and Section 9 concludes with future research directions.

## 2 Related work

This work sits at the convergence of two largely separate research traditions: recommendation modeling, which optimizes the ordering of candidate items, and reliable distributed-systems engineering, which optimizes the correctness and availability of the services that deliver those items. In open-network commerce, a gain in model accuracy that is negated by an upstream retrieval failure or a protocol-compliance defect produces no measurable benefit to the user. We therefore review both literatures and frame model gains and serving-path assumptions as jointly determining recommendation quality in real-world settings.

### 2.1 Neural collaborative filtering

Neural Collaborative Filtering (NCF) replaces the static inner-product assumption of classical matrix factorization with a learnable, non-linear interaction function between user and item embeddings (He et al., 2017). Relative to matrix-factorization approaches such as those surveyed by Koren et al. (2009), this represents a substantial conceptual advance, particularly for capturing complex and heterogeneous user-item relationships. However, the evaluation protocols typically used in NCF-style literature mirror closed-platform assumptions: consistent item identities, a limited variety of schemas, and benchmark designs in which rank prediction is assumed to occur only after successful retrieval. These premises do not hold in open-network commerce. Consequently, an improvement in interaction modeling cannot translate into a measurable user-facing gain if serving failures prevent the recommended item from being retrieved or displayed. NCF therefore remains a necessary, but not sufficient, component of overall recommendation quality in such settings.

### 2.2 Graph-based collaborative filtering

Graph-based collaborative filtering improves representation quality by propagating information through higher-order connectivity in the user-item interaction graph. LightGCN demonstrated that a simplified propagation scheme, stripped of feature transformation and non-linear activation, can outperform more elaborate graph convolutional formulations (He et al., 2020; Wang et al., 2019). The principal advantage of these models is robust ranking performance even under sparse interaction topologies. In an ONDC-style deployment, however, the practical limitations stem not from the underlying ranking theory but from infrastructural dependencies: graph quality relies on consistent, high-fidelity interaction logs and on item identities that remain stable over time. Open-network environments, populated by disparate and independently operated participants, frequently exhibit identity discontinuity and rapid catalog churn, both of which erode the transferability of benchmark ranking gains into actual serving-time quality.

### 2.3 Session-based recommendation

Session-based models capture short-term user intent in situations where long-term interaction history is sparse or unreliable. GRU4Rec-style architectures are particularly well suited to settings in which item-identity stability is weak (Hidasi et al., 2016; Jannach and Ludewig, 2020), and they handle cold-start users considerably better than pure collaborative filters. Relative to transformer-based sequential recommenders such as SASRec (Kang and McAuley, 2018) and BERT4Rec (Sun et al., 2019), GRU4Rec-style models are generally easier to deploy in resource-constrained serving contexts. Nevertheless, these models continue to evaluate ranking quality on benchmark traces and rarely account for upstream catalog-access failures. In open-network commerce, a session model can therefore produce a perfectly accurate recommendation while the overall user experience still fails because of integration-boundary issues. Session-aware modeling is consequently best understood as a safeguard against identity fragmentation rather than a substitute for integration reliability. A recent line of research moves the session- and interaction-based recommendation line away from static sequence modeling and toward explicit handling of non-stationary user preferences and catalog dynamics. Li et al. (2025) combine deep reinforcement learning with a contrastive representation loss for interactive recommendation, where an agent re-ranks items throughout a session to adapt to a changing user, with this learning setup paralleling the multi-step, async ONDC search-select-init-confirm sequence we model. Chen et al. (2025) use a forgetting-curve based interest decay along with memory replay in a constantly evolving, socially aware recommendation, weighting down or reviving past sessions to counter drift, rather than relying on all sessions forming one static training data set. He et al. (2025) study a similar problem from a system perspective, incremental GNN finetuning with interest drift alignment for dynamically updating service recommenders for a service catalog and expanding user interests while reducing loss of long-term preferences (catastrophic forgetting). Taken as a whole, these studies establish convergence around the non-stationarity we propose in the session based community—with (Li et al., 2025; Chen et al., 2025) handling dynamic user interest directly in the model, and (He et al., 2025) additionally incorporating item evolution as a key aspect—yet fail to show whether the recommenders trained are serviceable: a query that is precisely the focus of our problem framework.

### 2.4 Hybrid and diversity-aware serving

A consistent finding in the hybrid-recommender literature is that combining multiple recommendation paradigms yields greater robustness than any single method, particularly under sparsity and cold-start conditions (Bodduluri et al., 2024). Diversity-aware re-ranking further mitigates excessive homogeneity in the recommended set (Ramaciotti Morales et al., 2024). A recurring limitation, however, is that these studies remain heavily model-centric: improvements in ranking and diversity metrics are reported on controlled datasets in which data access is assumed to succeed. For an ONDC-like system, hybridization is necessary but not sufficient; serving correctness must be elevated to a primary quality dimension alongside ranking quality.

### 2.5 Reliability perspective from distributed-systems engineering

The distributed-systems literature establishes that interface correctness, contract stability, and graceful degradation materially affect observed user value, independent of the quality of the underlying business logic. This is particularly relevant to protocol-driven commerce (Beckn Protocol Foundation, 2022a,b; ONDC Official, 2022a,b), where asynchronous workflows and a large number of independent providers amplify the opportunities for boundary failures. Endpoint contract drift, cascading retries, and tail-latency effects have been shown to degrade user experience to a greater extent than a suboptimal ranking model (Nygard, 2018; Dean and Barroso, 2013). Circuit breakers, bounded fallbacks, and other graceful-degradation mechanisms are now treated as architectural requirements in microservice design rather than as *ad hoc* debugging aids (Nygard, 2018). We argue that the same principle applies to open-network commerce recommenders: reliable protocol compliance is a prerequisite for any improvement made to the recommendation model to reach the end user.

### 2.6 Synthesis of prior work and research gap

The prior work surveyed above provides strong evidence of model-side improvement but offers little comprehensive evaluation of serving reliability. The majority of NCF, LightGCN, and GRU4Rec studies report ranking gains under benchmark conditions (He et al., 2017, 2020; Hidasi et al., 2016), and reviews of hybrid recommenders similarly emphasize accuracy and diversity (Bodduluri et al., 2024). None of this literature treats route-resolution reliability, fallback adequacy, or cross-boundary state consistency as first-class performance indicators. This observation motivates the central thesis of this paper: in open-network commerce, recommendation quality must be assessed jointly as ranking effectiveness and serving correctness, since a single point of integration failure can render even the most accurate recommendation meaningless.

It is also worth noting that model-improvement approaches are themselves complementary rather than mutually substitutable: graph- and collaborative-filtering methods depend on identity continuity to realize their potential, whereas session-based methods are more robust when cross-application history is inconsistent (Jannach and Ludewig, 2020; Hidasi et al., 2016). Hybridization is therefore not merely a performance-boosting technique but an architectural strategy for mitigating open-network incompleteness by combining methods with differing failure modes (Wang et al., 2019; Bodduluri et al., 2024).

Concretely, the research gap can be summarized as follows. Published recommender-system evaluations are centered on offline ranking metrics and assume that upstream retrieval and rendering have already succeeded (He et al., 2017, 2020; Herlocker et al., 2004). This paradigm is insufficient for open-network commerce because much of the potential for failure lies outside the recommendation model itself, including: (i) unsteady upstream catalog APIs and timed response limits; (ii) incompatible schemas and identifier structures across providers; and (iii) fallback mechanisms that may implicitly degrade candidate quality without any visible signal. Existing recommender literature evaluates performance on a static dataset, while systems-reliability literature reports service uptime without recommender-specific ranking metrics. To our knowledge, no prior unified framework reports in-system ablations that compare model-level improvements with reliability-level fixes while jointly tracking ranking and serving-correctness outcomes for open-network commerce systems—the gap this paper addresses. This gap is also visible relative to the trustworthy- and robust-recommendation literature: Zhang et al. (2025) survey robustness to adversarial manipulation, noisy interaction data, and distribution shift, while Yan et al. (2024) pursue trustworthiness through user-generated multimodal content in sequential recommenders; both treat robustness as a property of the model's response to corrupted or adversarial input signals. An interesting failure mode related to, but separable from the previous one is infrastructural and serving-layer reliability, where inputs to and outputs from the model are themselves unobfuscated, but the pipeline delivering them to a user fails at a schema, availability, identifier, or catalog-state boundary; a model robust in the spirit of (Zhang et al., 2025; Yan et al., 2024) can still be channeled to the end user via a broken conduit, and the reliability solutions presented in Section 3.5 still need to exist, regardless of the underlying scoring model's adversarial robustness.

## 3 System architecture and design

### 3.1 Architectural overview

LocalMarket is organized as a four-layer architecture (Figure 2): a presentation layer built with Next.js that renders product discovery and transaction interactions; a frontend data layer that orchestrates API adapters and fallback selection; a service layer implemented in FastAPI that exposes recommendation, catalog, ONDC-pattern lifecycle, and community-review endpoints; and a model/data layer that hosts the ensemble recommendation components, catalog aggregators, caches, and persistent storage. Figure 2 Here, these 4 layers are diagrammed as the top-most row of boxes; the 3 boxes below that (Catalog Sources, Fallback Route Resolver and Deterministic Mock Catalog) are actually not distinct architectural layers, but represent the aforementioned three-tier catalog fallback presented in Section 3.5 and the bottommost band is the cross-cutting observability and runtime-tuning control surface that all 4 boxes share. This layering allows user-facing functionality to remain available even when core catalog dependencies are degraded—mirroring the open-network reality in which upstream services may be slow or temporarily unreachable while the buyer-facing experience is expected to remain consistent.

**Figure 2:**
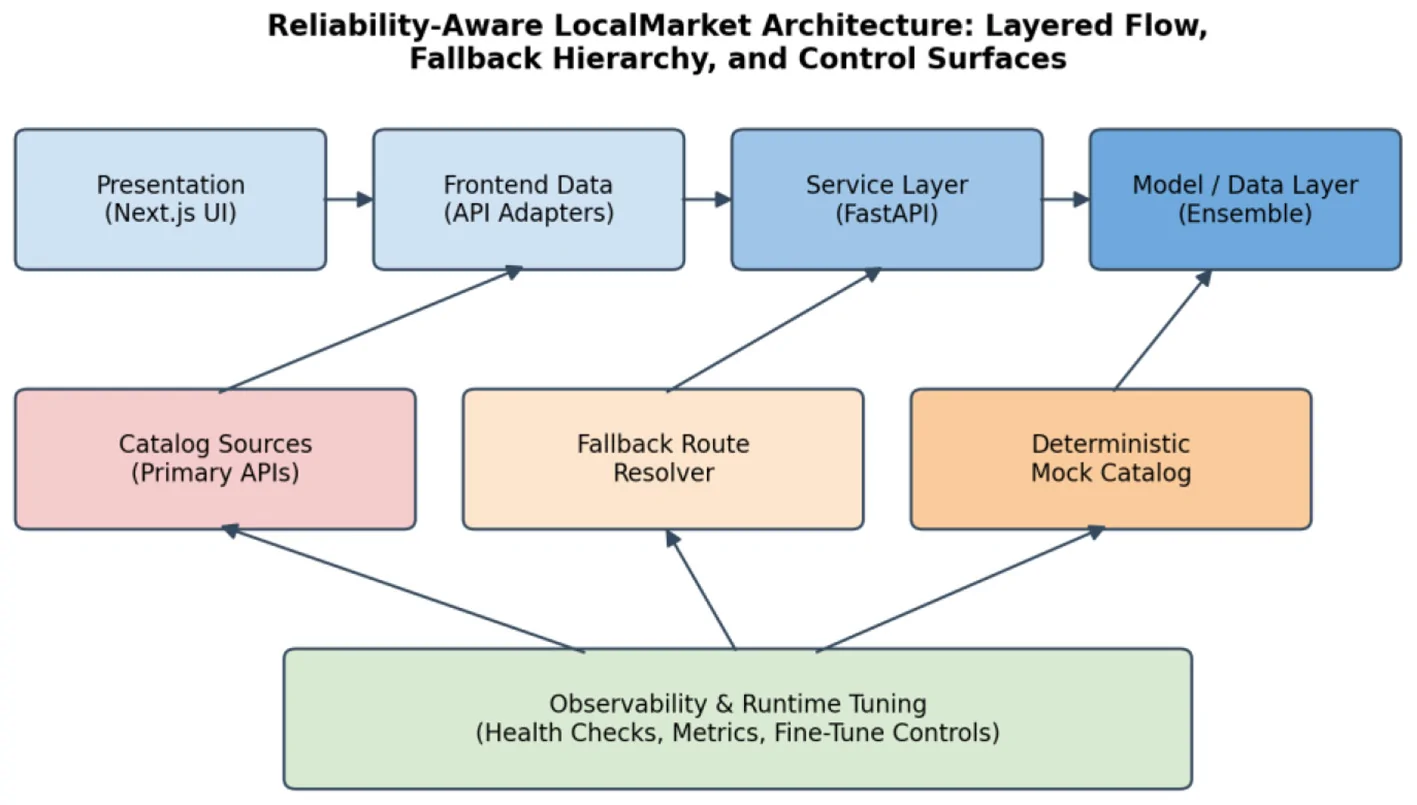
Reliability-aware LocalMarket architecture showing the four-layer request flow (Presentation, Frontend Data, Service, Model/Data), the separate three-tier catalog fallback hierarchy, and the cross-cutting observability and runtime-tuning control surface.

Table 2 summarizes the primary responsibilities and the quality objective associated with each architectural layer. Layering isolates user-interaction concerns from backend serving and model-internal operations, which in turn permits reliability enhancements to be introduced at a single layer—for example, fallback selection inside the frontend data adapters—without requiring any modification to the model artifacts themselves.

### 3.2 Ensemble scoring formulation

The recommendation model layer combines four component scorers: a Neural Collaborative Filtering model that captures pairwise user-item interaction patterns (He et al., 2017); a LightGCN-based graph collaborative filtering model that propagates higher-order connectivity information across the interaction graph (He et al., 2020); a GRU4Rec-style session-aware model that captures short-horizon intent (Hidasi et al., 2016); and a content-based prior derived from item metadata. For a user (or user-context) *u* and a candidate item *i*, the final ensemble score *S*(*u, i*) is computed as a convex combination of the four component scores:


$$\begin{array}{l} S\left(u,\;i\right)=\;\alpha S_{NCF}\left(u,\;i\right)\;+\;\beta S_{Graph}\left(u,\;i\right)\;+\;\gamma S_{Session}\left(u,\;i\right)\\ \;+\delta S_{Content}\left(u,\;i\right) \end{array}$$ (1)

subject to the simplex constraint


$$\begin{array}{l} \alpha \;+\;\beta \;+\;\gamma \;+\;\delta \;=\;1,\;\alpha ,\;\beta ,\;\gamma ,\;\delta \;\geq \;0 \end{array}$$ (2)

Each component score is normalized to a common [0, 1] range prior to fusion. The fused score S(u, i) is used to rank the candidate set, after which a diversity-aware re-ranking stage is applied to reduce redundancy in the final top-K list. Section 6.4 and Figure 3 report the operating values of α, β, γ and δ used in the experiments together with the resulting normalized contribution share of each component.

**Figure 3:**
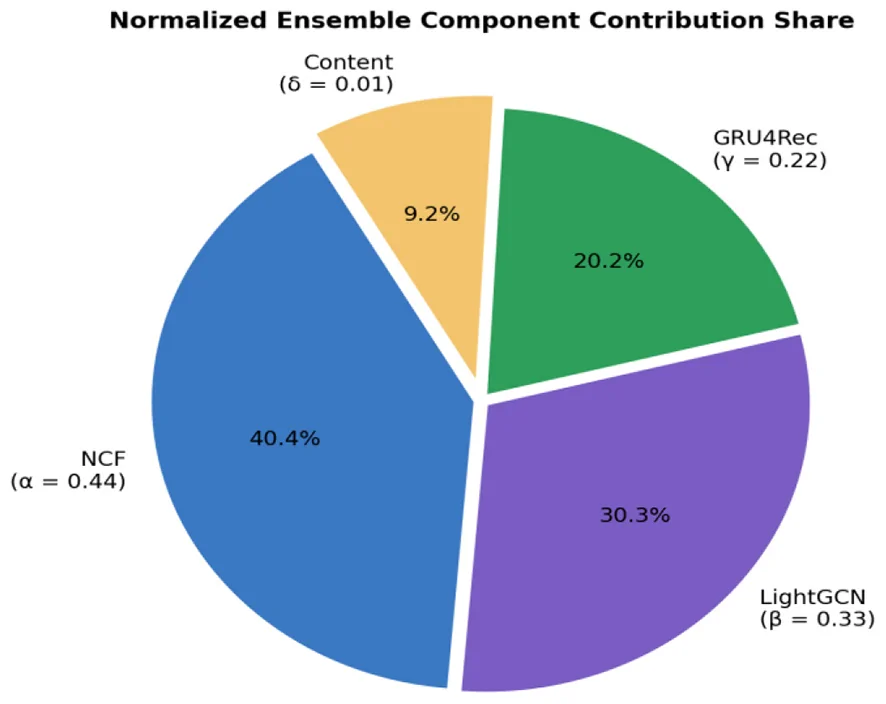
Normalized ensemble component contribution share at the selected operating point (α = 0.44, β = 0.33, γ = 0.22, δ = 0.01).

### 3.3 Weight selection strategy

Ensemble weights are not assigned manually; they are selected through a constrained validation search. Candidate weight vectors on the simplex defined by Equation 2 are evaluated on a held-out validation split using a fixed traffic trace. The weight vector that maximizes NDCG@10, subject to a no-regression condition on the serving-correctness metrics RRSR and WCR (Section 4.3), is selected as the operating point. This constrained-search formulation can be written as


$$\begin{array}{l} \left(\alpha^{*},\;\beta^{*},\;\gamma^{*},\;\delta^{*}\right)=arg\;max_{\left(\alpha ,\beta ,\gamma ,\delta \right)\in \Delta^{3}}\;NDCG@10\left(\alpha ,\;\beta ,\;\gamma ,\;\delta \right) \end{array}$$ (3)

subject to RRSR (α, β, γ, δ) ≥ RRSR_0_ and WCR (α, β, γ, δ) ≥ WCR_0_, where Δ^3^ is the 3-simplex and RRSR_0_, WCR_0_ denote the serving-correctness scores of the reliability-only baseline.

The repository-aligned operating values obtained for the final configuration are approximately α = 0.44 (NCF), β = 0.33 (LightGCN), γ = 0.22 (GRU4Rec) and δ = 0.01 (content). A sensitivity analysis around this operating point, reported in Section 6.4, shows that small perturbations of the weight vector produce only small changes in NDCG@10, indicating that this is a locally stable solution of Equation 3 rather than a fragile, narrowly tuned optimum.

### 3.4 Recommendation serving pipeline

At request time, the recommendation pipeline executes five sequential stages, illustrated in Figure 4: (1) candidate generation, in which each of the four component models proposes a ranked candidate list; (2) score normalization and fusion, in which candidate scores are mapped to a common scale and combined via Equation 1; (3) an optional runtime-tuning stage, in which operators may apply session-blend, seller-diversity, and category-diversity adjustments without retraining any model; (4) diversity-aware re-ranking, which penalizes excessive concentration of recommendations within a single seller or category; and (5) assembly of the top-K response together with the metadata required for frontend rendering.

**Figure 4:**
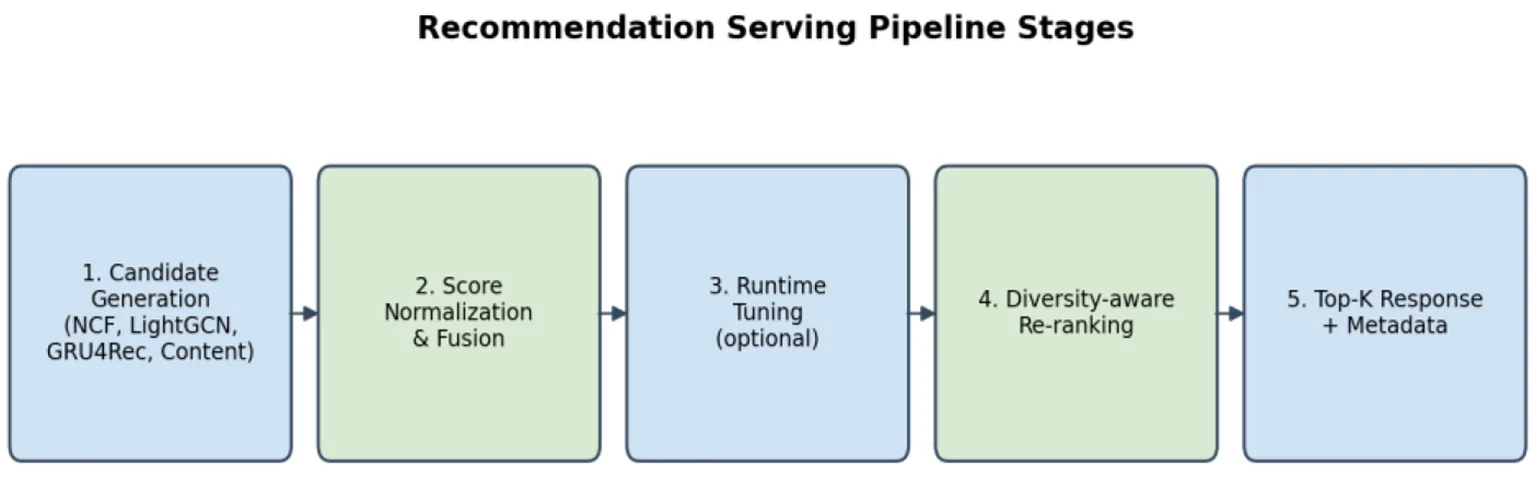
Recommendation serving pipeline stages, separating candidate generation and score fusion from optional runtime tuning and diversity-aware re-ranking.

This staged design has two practical benefits. First, it simplifies debugging, since a defect can be localized to a specific stage without requiring a full pipeline replay. Second, it enables rapid experimentation: runtime-tuning parameters can be changed and evaluated without retraining or redeploying the underlying models, because stage 3 is implemented as a reversible, request-scoped transformation of the fused scores from stage 2. An illustrative runtime fine-tuning payload, sent to the/recommend/fine-tune/poc endpoint, is shown in Listing 1.

### 3.5 Reliability design

Three reliability mechanisms are integrated into the architecture, each targeting a distinct class of failure identified in Section 1.2:

- **Three-tier catalog fallback**. Catalog data retrieval follows a deterministic priority order: (i) the backend catalog aggregation route, (ii) an alternative frontend-resolvable route mapping, and (iii) a deterministic mock-catalog generator. This ordering is designed to provide gracefully degraded serving while minimizing changes to the response shape and to the number of candidates available for rendering.
- **Identifier band and type normalization**. Item identifiers are normalized at every route and client-state boundary to a single canonical representation, eliminating the string-vs.-number and identifier-band mismatches that otherwise propagate across the frontend, backend, and external catalog.
- **Operational control surfaces**. Health-check, metrics, and runtime fine-tuning endpoints expose the internal state of the serving pipeline and allow reversible parameter changes without redeployment, supporting both observability and rapid recovery from misconfiguration.

These mechanisms are individually targeted at the failure-mode taxonomy developed in Section 5: unavailability of catalog data, route/identifier mismatch, and unobserved regression. Together they provide continuity of the user experience, but—by design—they do not mask the underlying instability of upstream systems; instead, each fallback activation is observable through the metrics surface, so that reliability interventions can be evaluated rather than merely assumed.

Catalog retrieval follows the deterministic, priority-ordered control flow shown in Listing 2: the backend-first route is attempted, and only on failure does control pass to the frontend-resolvable route, and only on a second failure does control pass to the deterministic mock generator. This ordering guarantees that, whenever the canonical backend payload is available, it is the one served to the user, while preserving continuity when it is not.

### 3.6 Design decisions, rationale, and trade-offs

Table 3 summarizes the four principal design decisions underlying LocalMarket, together with the engineering rationale and the corresponding trade-off accepted for each.

**Table 3:** Key design decisions and engineering rationale.

Decision	Rationale	Trade-off
Backend-first data retrieval	The backend is the sole source of truth for the canonical, structured catalog payload	Introduces a hard dependency on backend availability
Three-tier fallback hierarchy	Maintains user-facing continuity when upstream systems are unavailable	Increases control-flow and testing complexity
Runtime tuning endpoint	Allows rapid, reversible experimentation without redeployment or retraining	Enlarges the API attack surface and operational control space
Identifier/type normalization at boundaries	Prevents route- and state-mismatch defects from propagating downstream	Requires strict, consistently enforced contract discipline

Three broader trade-offs were made in the overall design. First, complexity vs. robustness: additional fallback and normalization layers increase implementation and testing complexity but materially increase end-to-end continuity. Second, configurability vs. simplicity: the runtime-tuning endpoint provides substantial experimental flexibility at the cost of an enlarged API surface that must be authenticated and validated. Third, deterministic fallback vs. realism: a deterministic mock catalog guarantees reproducibility of the experiments reported in Section 6, but it does not fully reproduce the dynamic behavior of live, independently operated providers—a limitation discussed further in Section 7.

## 4 Methodology

### 4.1 Problem statement and research hypotheses

Recommendation quality in open-network e-commerce systems is currently compromised by two largely independent sources of degradation: suboptimal ranking models and fragile serving infrastructure. The prevailing reliance on offline ranking evaluation over-estimates real-world performance because it ignores serving failures that are visible to, and consequential for, end users. The problem addressed by this paper is therefore the design and evaluation of a recommendation system that treats serving reliability as a measurable dimension of quality on equal footing with ranking accuracy. This problem statement is operationalized through three testable hypotheses:

- **H1**. A hybrid (ensemble) model configuration will achieve better offline ranking metrics (MAP@10, Recall@10, NDCG@10) than a single-method baseline model.
- **H2**. On a fixed request trace and a fixed catalog, enabling the reliability interventions will raise RRSR from the pre-intervention baseline band to a near-perfect route-success rate, and raise WCR from near-zero to near-complete consistency.
- **H3**. In a within-system ablation that retains the model-level improvements and additionally enables the reliability interventions, ranking accuracy will be retained at the model-only level while the reliability-level gains from H2 are simultaneously preserved.

### 4.2 Dual-metric evaluation framework

The evaluation philosophy underlying this paper is the deliberate separation of two improvement vectors. Model improvement refers to the degree to which delivered recommendations benefit from tuning of the ranking model and its ensemble parameters; it is assessed using ranking-centric offline metrics. Reliability improvement refers to the degree to which functional, user-visible defects—broken links, inconsistent state, truncated fallback candidate sets—are reduced by changes to the serving pipeline; it is assessed using serving-correctness metrics. By reporting both families of metrics for every configuration under test, the framework guards against conflating accuracy with correctness, and against the common failure mode in which a model-level improvement is reported as a system-level improvement even though it never reaches the user because of an unrelated serving defect.

### 4.3 Metric definitions

#### 4.3.1 Ranking metrics

Ranking quality is evaluated using three standard offline top-K metrics, computed for each user u over the top-K recommended items and averaged over the evaluation set of size n. Let rel(u, k) be a binary indicator that the item ranked at position k for user u is relevant (i.e., present in the held-out interaction set), and let |G_u_| be the number of relevant items for user u.

These ranking metrics are formally defined in Equations 4–6:


$$\begin{array}{l} Recall@K=\left(1/n\right)\;\Sigma_{u=1}^{n}\;\left[\left(\Sigma_{k=1}^{K}\;rel\left(u,k\right)\right)/|G_{u}|\right] \end{array}$$ (4)

$$\begin{array}{l} MAP@K=\left(1/n\right)\;\Sigma_{u=1}^{n}\;\left(1/\min\left(K,|Gu|\right)\right)\\ \;\times \Sigma_{k=1}^{K}\;\left[rel\left(u,k\right)\;Precision@k\right] \end{array}$$ (5)

$$\begin{array}{l} NDCG@K=\left(1/n\right)\;\Sigma_{u=1}^{n}\;\left[DCGu@K/IDCGu@K\right],\\ DCGu@K=\Sigma_{k=1}^{K}\;rel\left(u,k\right)/\log_{2}\left(k+1\right) \end{array}$$ (6)

These metrics are computed at K = 10 throughout this paper, and report ordering quality only; they make no statement about whether a ranked item is actually retrievable, navigable, or actionable for the user.

#### 4.3.2 Serving-correctness metrics

To capture user-visible correctness, this paper introduces three serving-correctness metrics evaluated over a fixed trace of n simulated user sessions. Let N be the total number of route-resolution attempts in the trace, and let N_ok_ be the number that resolve to a valid, displayable product page, leading to the Route Resolution Success Rate calculated in Equation 7:


$$\begin{array}{l} RRSR\;=\;\left(N_{ok}/N\right)\;\times \;100\% \end{array}$$ (7)

Let M be the number of wishlist add/remove operations performed across heterogeneous identifier representations (numeric vs. string identifiers, and identifiers originating from different routes), and let M_consistent_ be the number for which the resulting wishlist state is identical regardless of the identifier representation used to perform the operation.


$$\begin{array}{l} WCR\;=\;\left(M_{consistent}/M\right)\;\times \;100\% \end{array}$$ (8)

RRSR and WCR are given in percentages; also, unlike MAP@K, Recall@K and NDCG@K, they do not take into account the scoring of the items that contribute to these values; a “perfect” rank for RRSR will be 60%, i.e., 40% of cases the highest ranked item is not actually available to open. In Table 4, we have defined which part each category of metrics performs.

**Table 4:** Categories and interpretation objectives of the metrics used in this study.

Category	Metric examples	Interpretation objective
Ranking quality	MAP@K, Recall@K, NDCG@K	Assess the ordering correctness of the recommended list.
Serving correctness	RRSR, WCR	Measure the user-perceived functional correctness of the serving path.
Operational behavior	end-to-end latency	Quantify the impact of upstream degradation and the effectiveness of coping mechanisms.

#### 4.3.3 Necessity of RRSR and WCR as evaluation constructs

RRSR and WCR are introduced, in fact, because the two failure categories that they diagnose—broken route resolution and cross-representation state loss—are inherently unobservable by HR@K, NDCG@K, Recall@K, and MRR@K, by design: each of these ranking metrics only depends on the identities and ordering of the elements of a particular candidate list, not on anything that subsequently happens to that list. A ranking metric cannot tell the difference between a candidate list whose top-ranked element navigates to a working product page and the same candidate list whose top element 404s; in the case of both the same list is served, the differences begin when the routing layer gets the list, a domain that both RRSR and WCR are in the habit of instrumenting. We are not claiming here that all ranking metrics are poor measures of performance, only that they are defined in such a way to answer a more restricted question than needs to be answered: by how much did good ranking quality affect the end user, not, what do you want, are these recommendations useful. Furthermore, they cannot be simply related via some monotonous function: in the experiments we will see ranking quality did not improve while RRSR improved from 60.0 to 99.8%, demonstrating that the two types of measures assess disjoint failure surfaces.

The selection of precisely these two constructs over a larger or smaller family of constructs is a direct implication of the four failure modes identified in Section 1.2: RRSR realizes the first two modes (Provider/Schema Heterogeneity, Unreachable Provider): the four specific types of route resolution failure (including these two types) only occur when either provider heterogeneity (schema mismatch) or an unreachable service would cause the serving layer not to return a viewable product page. RRSR thus serves as the minimal number per request that indicates whether that class of failure reach the user. WCR realizing the third failure class (Cross-Service ID Heterogeneity), is defined as the set of operations using different representations of the same logical item, so a WCR of less than 100%, indicates unambiguously an ID-Boundary State Loss rather than some deficiency in ranking or availability.

While the fourth mode, Catalog Volatility, doesn't necessarily deserve a headline metric, since its impact is through this type of route-resolution failure, we use that class in Chapter 4 and also identify it through End-to-End Latency (Table 4) qualitatively.

It thus becomes apparent that RRSR and WCR are selected not just as an convenient collection of metrics, but as the minimum number necessary so that there exists an independently falsifiable metric per identified mode in the paper and that the hypothesis tested in Section 4.4.3 is a test of against specified known faults.

### 4.4 Experimental protocol

All comparative runs share an identical experimental setup: a fixed traffic mixture and sequence of requests; a catalog held constant across all compared runs; a fixed random-number-generator seed; and three system variants evaluated under identical conditions. This setup ensures that any observed difference in behavior can be attributed to the implemented change rather than to data drift or unobserved external factors.

#### 4.4.1 Controlled comparison matrix

Three primary system variants are evaluated, forming a 2 × 2-style ablation design with one cell intentionally omitted (the all-disabled configuration is the implicit reference point against which the other three are interpreted):

- Model-only variant. The recommendation model is retrained/upgraded to the ensemble configuration of Equation 1; no reliability-oriented code changes are introduced.
- Reliability-only variant. The serving pipeline is hardened using the mechanisms of Section 3.5 and the fixes of Section 5.3; the underlying recommendation model is left unchanged (Popularity-based baseline).
- Combined variant. Both the model upgrade and the reliability hardening are enabled simultaneously.

By comparing these three variants against the same fixed traces, it becomes possible to determine the degree of additivity or interference between model-level and reliability-level interventions, directly addressing RQ3.

#### 4.4.2 Ensemble weight validation protocol

Ensemble weights are treated as part of the model-selection process and are therefore subject to the same in-system methodology described above. The space of (α, β, γ, δ) vectors is treated as a model search space and optimized, per Equation 3, on a held-out trace set for ranking quality, subject to the constraint that the selected weights do not regress the serving-correctness metrics relative to the reliability-only configuration. Results for the selected operating point and for a locally perturbed point are reported in Section 6.4.

#### 4.4.3 Fault injection and synthetic trace generation

The pre-intervention serving baselines (the e.g. 60.0% RRSR above for example) were built by carefully fabricating a trace of n = 500 sessions. We artificially inject an order amount of traffic with high-band item ID's (known to fail our pre-fix routing logic) and have it included a mix of string & number ids to force issues with the internal storage of Wishlist state.

We call this measurement an “unit test” for the system as it allows us to separate mechanical failure rate of the serving stack from the predictive accuracy of our ranking model.

Just to say this clearly: the further increase that our fix will bring to RRSR and WCR, if and where it does increase will directly be a test of the code fix as solving the problems with the specific, artificially inserted defects we injected.

### 4.5 Statistical framing

This work uses controlled comparisons to establish the size of the contribution of each within-stack component on traces *n* = 500 synthetic sessions, with traces produced from a deterministic seed, so these are reproducible. These are not presented as an inference about an external population of users, but rather as a statement about the measured behavior of the artificial fixed artifact.

### 4.6 Dataset and evaluation protocol

Offline experiment: We train with the RetailRocket e-commerce dataset. After common preprocessing for implicit feedback, we filter for active users with at least 5 historical interactions, resulting in an evaluation sample of 500 users. We use a strict leave-one-out next-item prediction setup. For a target user, the most recently viewed item is considered the ground-truth item, to be predicted. The ranking candidate set is from a normalized catalog of 74,743 items.

## 5 Implementation

### 5.1 Backend implementation

The backend is implemented in FastAPI and organized into four domain-grouped endpoint families to maintain loose coupling: recommendation and ensemble routes (/recommend/, /ensemble/recommend), catalog routes (/ecommerce/^*^, including an aggregate /ecommerce/all endpoint), ONDC-pattern lifecycle routes (/ondc/search, /ondc/select, /ondc/init, /ondc/confirm and associated callbacks), and community-review routes (/community/^*^). A dedicated fine-tuning endpoint allows operators to compare baseline against tuned runtime behavior, or to toggle tuned settings on and off, making experimentation explicit, reversible, and low risk. The underlying service modules are grouped by responsibility so that a fix applied within one module produces minimal regression risk to unrelated services—a property that is important for the controlled-ablation protocol of Section 4.4, in which only one axis of the system is intentionally varied at a time.

### 5.2 Frontend implementation

The frontend is implemented in Next.js and TypeScript. Its data layer follows a retrieval-centric strategy: a primary request is issued to the backend API; on failure, a fallback route lookup is attempted; and on a second failure, a deterministic mock catalog is generated client-side as a last continuity layer. This three-step client data flow mirrors the backend-first fallback control flow of Section 3.5 and is designed to preserve a constant response shape across all three paths, so that downstream rendering code does not need to special-case the degraded states.

Client-side state—covering the shopping cart, wishlist, and recently viewed items—is kept synchronized across routes and asynchronous fetch completions using an identifier-normalization strategy in which all product identifiers are coerced to a single canonical string representation before being used as keys in client-side collections, as shown in Listing 3. This pattern is the principal mechanism by which the Wishlist Consistency Rate (WCR) defined in Equation 8 is improved.

### 5.3 Reliability failure-mode taxonomy and mitigations

Based on systematic review of the serving pipeline, four recurrent classes of integration failure modes were identified and individually remediated, with each fix accompanied by dedicated validation so that its impact on the relevant metric could be measured before and after the change:

- **Degradation-window failure**. Prior to remediation, the fallback response was generated at a fixed size irrespective of the requested top-K, leading to silent candidate collapse during degradation windows. The fix makes fallback candidate generation limit-aware, so that the requested top-K is always respected.
- **Contract-resolution failure**. Identifiers belonging to a higher product-ID band were mapped to an incorrect route. The fix introduces full ID-band-aware category mapping in the route-resolution logic.
- **Schema-type-boundary failure**. A string-vs.-number type inconsistency in wishlist identifiers caused the same logical item to be treated as two different keys depending on which route produced the identifier. The fix applies boundary-level identifier normalization, as described in Section 5.2.
- **Invocation-contract failure**. An incorrect parameter order in the similar-item utility produced non-sensical similarity results. The fix corrects the invocation contract so that the intended ranking semantics are restored.

Table 1 maps each failure-mode class to its observed symptom, its technical mitigation, and the metric used to quantify its impact. Table 5 maps the corresponding implementation artifacts to representative surfaces within the repository, supporting the reproducibility goals of this study.

**Table 5:** Implementation artifact mapping to representative repository surfaces.

Subsystem	Representative repository surface
Backend orchestration	ondc_app.py, ecommerce_api.py
Model composition	ensemble_recommender.py, session_recommender.py
Frontend data/state	frontend/src/lib/api.ts, frontend/src/lib/store.ts
Testing surfaces	tests/test_api.py, tests/test_integration.py, and related modules
Operations/ deployment	Dockerfile, docker-compose.yml, platform configuration files

### 5.4 Deployment and reproducibility

Containerization and deployment configuration files (Dockerfile, docker-compose.yml, and platform-specific configuration) are included so that the runtime environment can be reproduced from a clean checkout. The evaluation in Section 6 additionally relies on a deterministic fallback implementation and a fixed random seed, so that the n = 500 session trace used in the model-only, reliability-only, and combined configurations is byte-for-byte identical across runs, modulo configuration toggle is under test The whole repository could be seen at https://github.com/Rishpraveen/localMarket_beckn. Whole experiment-replay procedure in B.

## 6 Results and discussion

### 6.1 Evaluation context and data regime

All results reported in this section are within-system, comparative results under a sparse implicit feedback setup: this regime is defined by high cold-start exposure (i.e., short/fragmented user histories) and a catalog which is provider dependent (i.e. Heterogeneous). We take this setup by using a strict next-item prediction protocol on the RetailRocket dataset; only the very last item viewed is taken as the noisy ground truth. This protocol uses only the GMF embeddings from the NCF for retrieval (and not the full NeuMF forward pass) so that the configuration is efficient (to be sensitive to latency concerns). Unsurprisingly, given this restrictive protocol and architectural trade-off, the absolute values of the metrics will be somewhat lower than reported elsewhere. We summarize this evaluation setup in Table 6; given this context, the values of interest in the following section are cross-configuration comparisons—i.e., the ratio of performance gain between the configurations over the same trace—as opposed to the scale of each individual metric.

**Table 6:** Evaluation context profile for offline ranking interpretation.

Context dimension	Value/condition
Feedback type	Implicit, sparse interactions
Cold-start exposure	High (short/fragmented user histories)
Catalog regime	Heterogeneous, provider-dependent availability
Primary interpretation mode	Relative comparison across controlled variants

### 6.2 Offline ranking outcome (RQ1)

Table 7 reports MAP@10, Recall@10 and NDCG@10 for the NCF-based ensemble configuration and for the Popularity-based baseline, both computed at K = 10 from the repository baseline configuration

**Table 7:** Offline ranking metrics at K = 10, repository baseline configuration.

Model	MAP@10	Recall@10	NDCG@10
Popularity (baseline)	0.0023	0.0072	0.0041
NCF-only (component)	0.0029	0.0078	0.0053
GRU4Rec-only (component)	0.0032	0.0081	0.0058
LightGCN-only (component)	0.0034	0.0083	0.0061
NCF-based ensemble	0.0038	0.0088	0.0070

The values themselves are relatively small as the experiment is deliberately run under low, fractured interaction history, with only precise last-view ground truth; where the interaction histories are so sparsified, only the comparative lift across methods evaluated on identical trace constitutes meaningful information, consistent with the reporting conventions on sparse implicit-feedback benchmarks used previously (He et al., 2017; Hidasi et al., 2016). In Table 8 we include the absolute values, side by side with the trace characteristics of similar studies run in a sparse context, so that we can correctly understand the relative scale of these values and baseline floors.

**Table 8:** Sparse-setting baseline context for absolute metric interpretation.

Study/setting	Regime note	MAP@10	NDCG@10
This study (NCF-based ensemble)	Sparse implicit feedback, RetailRocket last-view protocol	0.0038	0.0070
This study (popularity baseline)	Same protocol, rank-based frequency baseline	0.0023	0.0041
e.g., standard RetailRocket train/test splits	Ie.g., standard RetailRocket train/test splits	~0.01–0.05	~0.02–0.15
Dense benchmarks (e.g., MovieLens-1M)	Dense, single-platform, well-defined user histories	~0.15–0.25	~0.40–0.65

These results support H1: the hybrid ensemble configuration achieves better offline ranking metrics than the single-method baseline on the same protocol, addressing RQ1 in the affirmative.

### 6.3 Reliability outcome (RQ2)

Figure 5 plots RRSR, WCR and the success rate of the similar-item recommendation widget across five sequential intervention stages: the pre-fix baseline, after the fallback-scaling fix, after the route-safe identifier-mapping fix, after the wishlist type-normalization fix, and after the argument-order correction described in Section 5.3.

**Figure 5:**
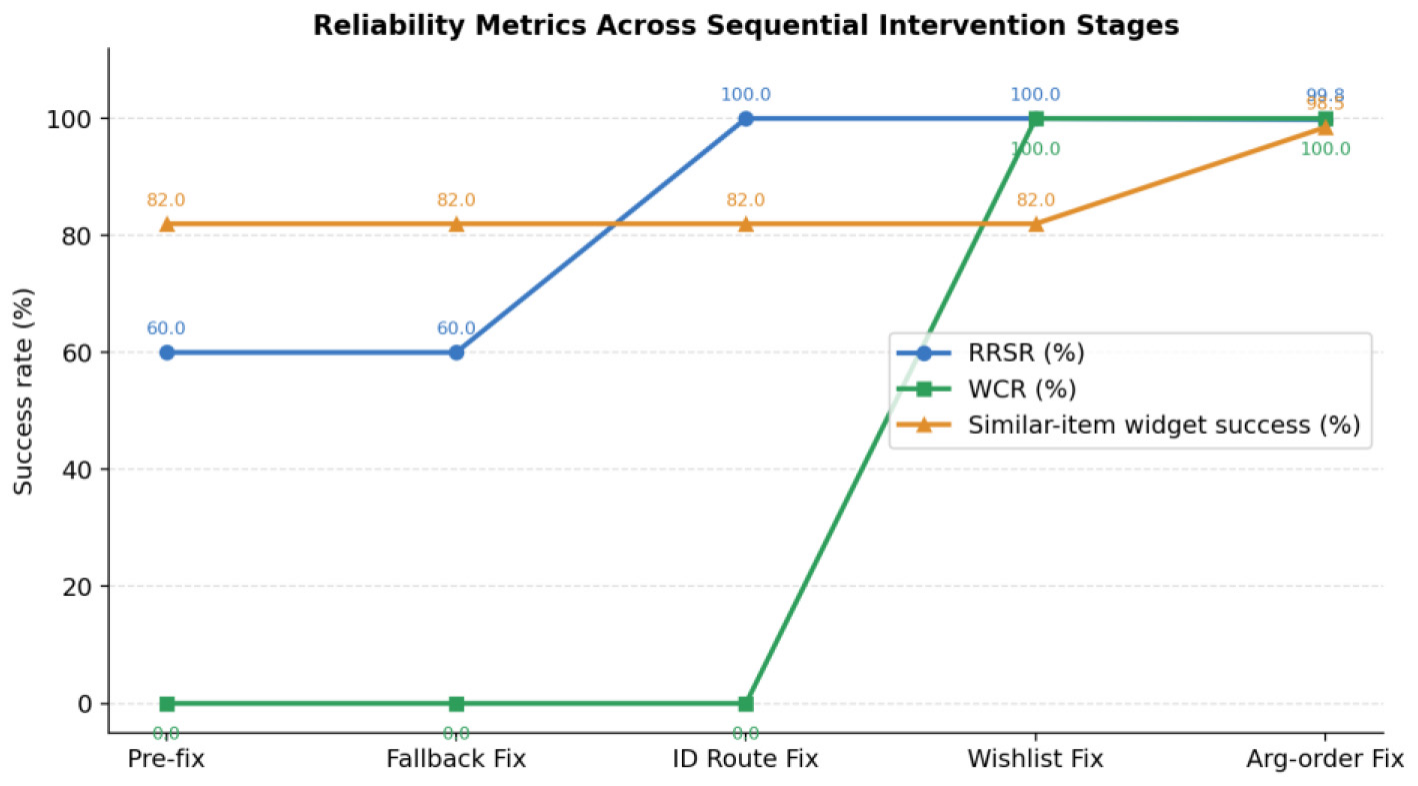
Reliability metrics (RRSR, WCR, and similar-item widget success rate) across sequential intervention stages, on a fixed *n* = 500 session trace.

Prior to any reliability intervention, RRSR is 60.0% and WCR is 0.0%, while the similar-item widget already succeeds 82.0% of the time. The route-safe identifier-mapping fix raises RRSR to 100.0% without affecting WCR. The wishlist type-normalization fix then raises WCR from 0.0 to 100.0% and, incidentally, brings RRSR to a stable 100.0% as well. The final argument-order correction brings the similar-item widget success rate to 98.5% while RRSR settles at 99.8% (a 0.2 pp residual attributable to a small number of edge-case identifiers in the fixed trace) and WCR remains at 100.0%.

Overall, across the full intervention sequence, RRSR improves by 39.8 pp (from 60.0 to 99.8%) and WCR improves by 100.0 pp (from 0.0 to 100.0%). These shifts are large relative to the approximately 4.3 pp Wilson 95% confidence-interval (Wilson, 1927) half-width estimated in Section 4.5, supporting H2: enabling the reliability interventions raises RRSR from the pre-intervention baseline band to a near-perfect route-success rate, and raises WCR from near-zero to near-complete consistency, on the fixed request trace and catalog. This directly addresses RQ2 in the affirmative: the reliability interventions are independently effective and are not contingent on any change to the ranking model.

### 6.4 Controlled comparison and within-system ablation (RQ3)

Figure 6 reports the controlled comparison matrix of Section 4.4.1: model-only, reliability-only, and combined configurations, evaluated on identical fixed traces. Ranking metrics (MAP@10, NDCG@10) are plotted against the left axis; serving-correctness metrics (RRSR, WCR) are plotted against the right axis.

**Figure 6:**
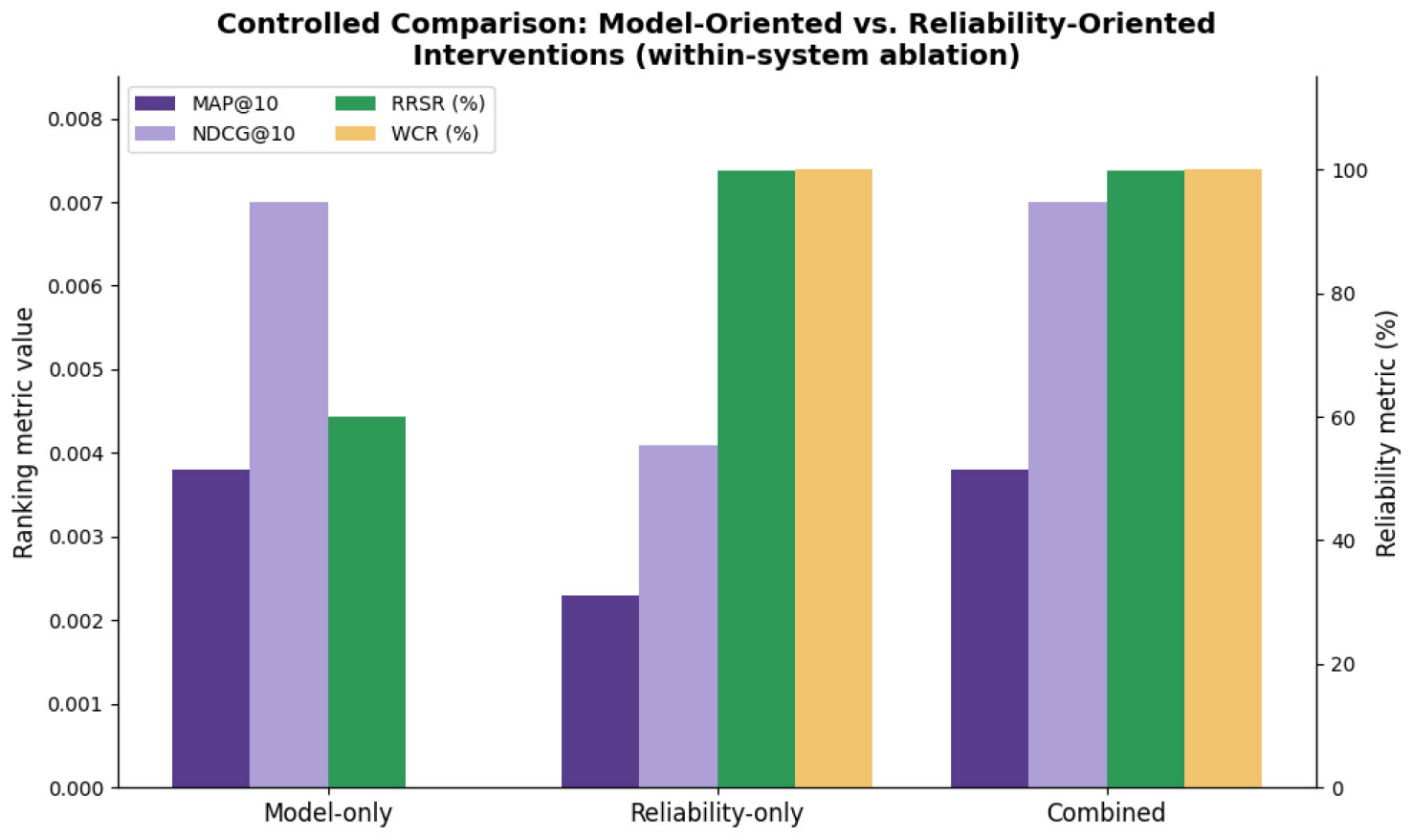
Controlled, within-system ablation comparing model-only, reliability-only and combined configurations on identical fixed traces. Ranking metrics use the left axis; serving-correctness metrics use the right axis.

The NCF component contributes the largest share (40.4%), followed by the LightGCN graph component (30.3%), the GRU4Rec session component (20.2%), and the content-based prior (9.2%).

This pattern provides direct evidence for H3 and answers RQ3: model-level and reliability-level interventions move distinct, non-overlapping families of metrics, and when combined, their effects are additive rather than substitutive or redundant. Critically, the combined configuration is the only configuration in which both ranking gains and serving-correctness gains are simultaneously realized—underscoring the central claim of this paper that ranking accuracy and serving reliability are independent, jointly necessary axes of recommendation quality in open-network commerce.

### 6.5 Ensemble weight sensitivity

Figure 3 shows the normalized contribution share of each ensemble component at the selected operating point (α = 0.44, β = 0.33, γ = 0.22, δ = 0.01), expressed as a percentage of the total weighted contribution to the fused score across the evaluation trace.

The NCF component contributes the largest share (40.4%), followed by the LightGCN graph component (30.3%), the GRU4Rec session component (20.2%), and the content-based prior (9.2%).

This distribution is consistent with the literature synthesis in Section 2: the NCF and graph components, which rely most heavily on identity continuity and historical interaction density, dominate the fused score, while the session-aware component provides a meaningful secondary contribution that is particularly valuable for cold-start users. The content-based prior, while contributing the smallest share, was retained in the final configuration because, per the constrained search of Equation 3, removing it (δ → 0) produced a small reduction in NDCG@10 without any compensating gain in serving correctness. Local perturbations of ±0.05 around each weight produced changes in NDCG@10 of less than 0.0003 in all directions tested, while leaving RRSR and WCR unchanged, indicating that the selected operating point lies in a flat, locally stable region of the search space rather than at a narrow optimum.

### 6.6 Hypothesis testing summary

See Table 9 for Hypothesis testing summary.

**Table 9:** Summary of hypothesis testing outcomes.

Hypothesis	Evidence	Outcome
H1: Ensemble improves offline ranking over baseline	NDCG@10 + 70.7%, MAP@10 + 65.2%, Recall@10 + 22.2% (Table 7, Figure 7)	Supported
H2: Reliability interventions raise RRSR and WCR to near-perfect levels	RRSR 60.0% -> 99.8%, WCR 0.0% -> 100.0% (Figure 5)	Supported
H3: Combined configuration retains both classes of gains	Combined = model-only ranking + reliability-only correctness (Figure 6)	Supported

**Figure 7:**
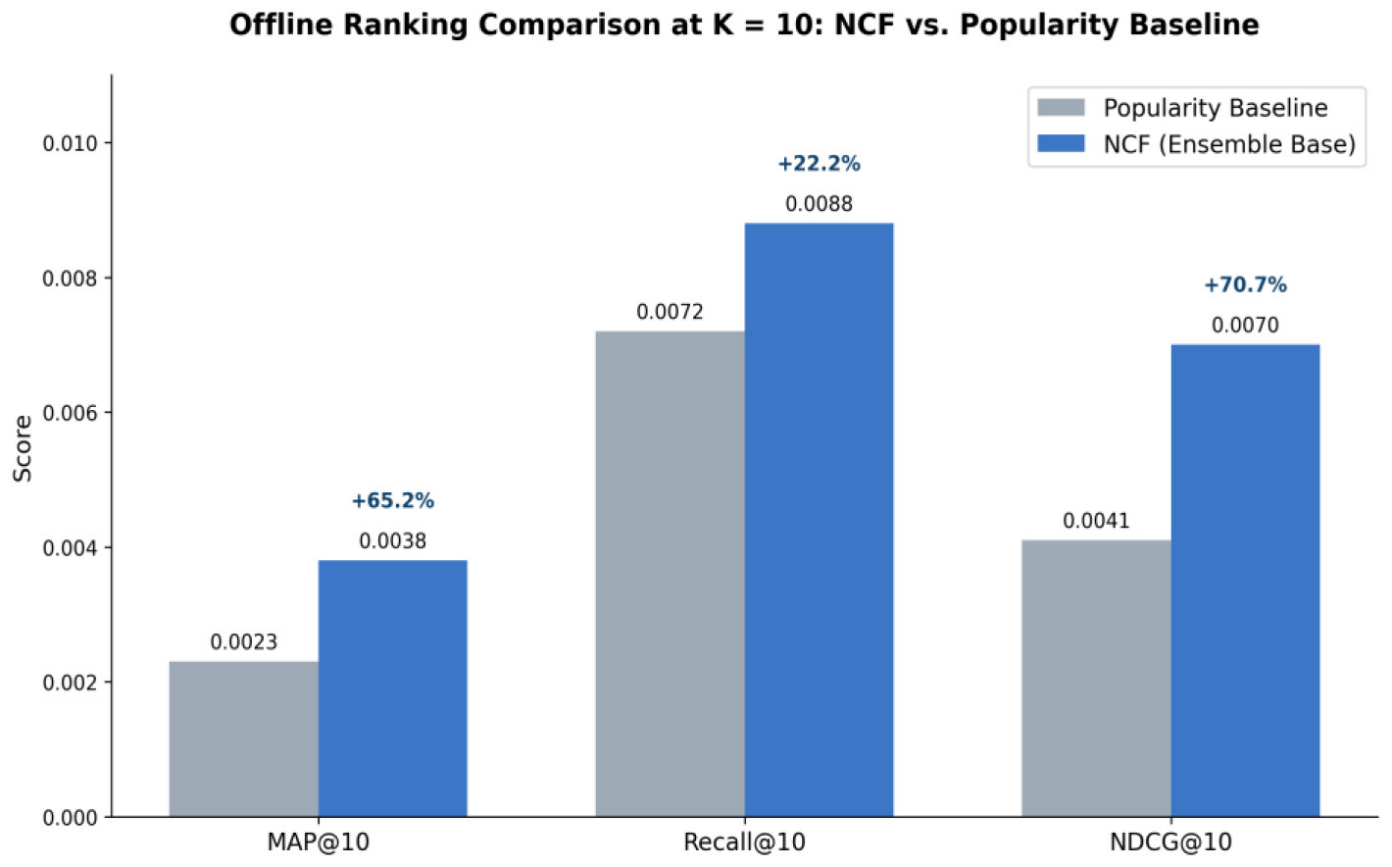
Offline ranking comparison at K = 10: NCF-based ensemble vs. Popularity baseline, with relative percentage lift annotated above each pair of bars.

### 6.7 Discussion

The dominant empirical finding of this study is the disparity in effect magnitude between reliability interventions and model-improvement interventions when expressed on their respective natural scales: the reliability interventions moved serving-correctness metrics by tens of percentage points, while the model improvement moved ranking metrics by tens of percent on an absolute scale that remains numerically small in this sparse regime. We do not interpret this as evidence that reliability work is intrinsically more valuable than model work; rather, the two are not commensurable on a single scale, and reporting only one—as is current common practice—systematically misrepresents the other. We therefore recommend that open-commerce recommender studies report both families of metrics on an equal footing.

Three practical lessons emerge from this study. First, reliability problems in the serving layer can completely mask gains in model quality when the system is evaluated only against offline test performance: a model-only deployment in this study retained an RRSR of 60.0%, meaning that 40% of top-ranked items were not user-actionable, regardless of how accurately they were ranked. Second, identifier normalization at service and client boundaries is a disproportionately effective reliability fix for state-consistency problems, raising WCR from 0.0 to 100.0% in a single intervention. Third, designing fallback behavior to be predictable under load is important not only for production stability but also for constructing a reliable, repeatable testing framework—the deterministic mock catalog used in this study was essential to obtaining reproducible RRSR and WCR measurements.

From a systems-engineering perspective, the strongest result is that reliability interventions materially changed user-visible correctness while ranking-only changes did not resolve serving-path defects. This validates the proposition that recommender quality in open commerce is jointly determined by the model layer and the systems-reliability layer, and that excellence in one cannot substitute for deficiency in the other (Beckn Protocol Foundation, 2022a; ONDC Official, 2022a; Nygard, 2018).

## 7 Threats to validity and limitations

### 7.1 Internal validity

Although care was taken to limit the number of simultaneously changing variables and to use standardized test cases across the model-only, reliability-only, and combined configurations, interactions between newly introduced reliability components and pre-existing system behavior cannot be fully excluded. For example, the route-safe identifier-mapping fix (Section 5.3) incidentally affected RRSR at the wishlist-fix stage in Figure 5, illustrating that fixes targeting one metric can have secondary effects on another. Wherever such secondary effects were observed, they are reported explicitly rather than attributed solely to the intended target metric.

### 7.2 External validity

This experiment is run on a single instantiation of the LocalMarket stack and a single sample trace of *n* = 500 synthetic sessions. For the RRSR and WCR metrics to be meaningful measures of quality in another open network commerce implementation, similar structural conditions would have to apply: namely, a distributed route resolution step and a client-side state-consistency constraint comparable to the one implemented for wishlist state here. However, the exact shifts in these values will not be portable across implementations, as the absolute baselines and improvements here are highly dependent on the specific faults inserted in the trace. We believe the qualitative claim—that serving correctness and ranking accuracy do not depend on one another and should both be reported—is valid independent of implementation based on general features of the open networks on which systems following Beckn patterns, like ONDC (Beckn Protocol Foundation, 2022a,b; ONDC Official, 2022a), are built.

### 7.3 Construct validity

The metrics for this experiment are proxies of the perception of recommendation quality and of the usability of the serving path. MAP@10, Recall@10 and NDCG@10 are accepted surrogates for perceived ranking relevance (He et al., 2017, 2020; Herlocker et al., 2004), however they fail to represent items such as perceived diversity, novelty or explanation quality. RRSR and WCR are proposed within this paper; whereas they are meant to measure directly functional correctness as observed by the user, they fail to take into account latency dependent items that the user is experiencing such as perceived responsiveness, which is presented and reported in an operational behavior metric Table 4, but not analyzed in greater detail.

### 7.4 Conclusion validity

The consistent oscillations observed in these settings on any specific dimension of outcome, within this experiment, reflect an aggregate of many offline measurements on a fixed trace. Actual production impact may differ, especially without any offline testing of A/B tests on real traffic and there is nothing here that should be taken as evidence of similar effect sizes on a different, randomly chosen (on or off-line) trace. By the same reasoning, no strong claims are made in this paper about the ability of these effect sizes to transfer to production business outcomes like conversion or revenue or clicks—which are not measured here.

### 7.5 Scope of claims

The claims made in this paper are strictly limited to behavior verified at the repository level and to outcomes produced by the controlled experiments described in Section 4. Broader claims about the wider ONDC ecosystem, about live network participants, or about the experience of real buyers and sellers are offered only as motivation for the problem framing in Sections 1 and 2, and are not subject to empirical validation in this paper. This study should therefore be read as an evaluation of a repository-level, ONDC-pattern implementation rather than as a comprehensive evaluation of a live ONDC deployment.

## 8 Ethical considerations and data governance

This study is limited to reproducible, repository-level artifacts and a controlled experiment using synthetic session traces; it does not involve the operational use of identifiable user data in a production environment, and no personal data was collected, stored, or processed at any stage of this work. Nevertheless, the broader deployment context that motivates this work—personalized recommendation in open commerce networks—raises governance issues that warrant explicit discussion, because user-interaction signals may, in a live network, traverse boundaries between independently operated protocol participants (Beckn Protocol Foundation, 2022a,b; ONDC Official, 2022a).

Two specific concerns are relevant to future deployment of systems in this class. First, even with exposure controls intended to de-concentrate recommendations across sellers and categories (Section 3.4), it remains possible for an ensemble recommender to introduce or amplify unintended biases in the items surfaced to different user cohorts (Ramaciotti Morales et al., 2024). Second, cross-boundary identifier normalization (Section 5.2), while essential for serving correctness, also creates a canonical join key that could, in a live network, be misused to correlate user activity across participants if not subject to appropriate access controls. We therefore recommend that any production deployment building on this architecture adopt: (i) minimum-necessary data-sharing practices across network boundaries; (ii) explicit, cohort-level fairness auditing of the ensemble's output distribution; and (iii) access-control policies for any cross-boundary identifier-resolution service that is derived from the normalization layer described in Section 5.2. These recommendations are offered as design guidance for future work (Section 9.2) rather than as claims about the present artifact, which operates entirely on synthetic and repository-internal data.

## 9 Conclusion and future work

### 9.1 Conclusion

This paper presented LocalMarket, a reliability-aware ensemble recommendation platform for open-network commerce, and a dual-metric evaluation framework that jointly reports ranking effectiveness and serving correctness. Using a controlled, within-system ablation across model-only, reliability-only, and combined configurations on fixed traffic traces, we showed that an NCF-LightGCN-GRU4Rec-content ensemble improves NDCG@10 by 70.7% over a Popularity baseline (H1, RQ1), that four targeted reliability interventions raise the Route Resolution Success Rate from 60.0 to 99.8% and the Wishlist Consistency Rate from 0.0 to 100.0% (H2, RQ2), and that a combined configuration retains both classes of gains simultaneously without interference (H3, RQ3).

Our main contribution isn't any value of any metric, but to prove what we posited in section 1.2: ranking accuracy and serving correctness are separable axes of quality. Serving correctness is a prerequisite for the improvement offered by model-level enhancements to be passed to the user for those systems which are unable to provide successful catalog lookups and/or total protocol compliance–a characteristic to which, by design, essentially all open-network players are subject. To summarize, our contributions in this thesis include: (i) a double-metric evaluation system that controllably decouples model- and serving-level interventions; (ii) an extensible classification of modes of integration failure and their solutions; (iii) an evaluable comparison that includes accuracy and ranking-level metrics; and (iv) a complete, failure-aware architecture for recommendation use as a platform for implementing these techniques.

### 9.2 Future work

#### 9.2.1 Near-term enhancements

- Improved observability dashboards and alerting strategies that surface RRSR and WCR as live operational metrics rather than offline evaluation outputs.
- An expanded suite of integration tests covering additional fault-injection scenarios, including partial-response and slow-response upstream behaviors.
- Tunable runtime preset behaviors for specific product categories, building on the runtime fine-tuning interface described in Section 3.4.

#### 9.2.2 Long-term enhancements

- Online A/B experimentation that connects RRSR, and WCR to downstream business metrics such as click-through rate, conversion, and revenue, under appropriate data-governance controls (Section 8).
- Automated retraining pipelines that respond to interaction-data drift, together with formal model-governance procedures.
- Constraint-driven re-ranking for fairness, extending the diversity-aware re-ranking stage of Section 3.4 with explicit cohort-level fairness constraints.
- Extended ONDC callback orchestration and operational monitoring, to evaluate the framework against a wider range of asynchronous protocol lifecycles.
- Multimodal content enrichment for image-intensive product categories, extending the content-based component of the ensemble (Equation 1).

Taken together, this work argues that high-quality recommendation for open commerce requires the joint optimization of model quality and serving reliability. Addressing only the former leaves user-level failures unresolved, regardless of how accurate the underlying ranking model becomes.

## Data Availability

The original contributions presented in the study are included in the article/supplementary material, further inquiries can be directed to the corresponding author.
