# Supplementary material for: Reliability-aware ensemble recommendation for open-network commerce: a dual-metric framework for jointly evaluating ranking accuracy and serving correctness
Source: Front Big Data. 2026 Oct 8;9:1911540. doi: 10.3389/fdata.2026.1911540 (PMC13686825; doi:10.3389/fdata.2026.1911540)
Supplement: Supplementary file 1 [file Supplementary_file_1.docx]

**Appendix A. Sample Implementation Code**

This appendix lists representative code excerpts referenced in Sections 3 and 5. Listings are reproduced from the LocalMarket repository in simplified form for illustration.

**Listing 1. Runtime tuning payload sent to the fine-tuning endpoint.**

JSON

POST /recommend/fine-tune/poc?user_id=1000&n=10
{
 "session_blend": 0.35,
 "min_sellers": 4,
 "min_categories": 3,
 "seller_bonus": 0.10,
 "category_bonus": 0.08,
 "apply": false
}

**Listing 2. Backend-first, three-tier catalog fallback control flow.**

Python

products = fetch_backend_all(limit_per_category=q)
if not products:
 products = fetch_frontend_route(limit=q)
if not products:
 products = deterministic_mock(limit=q)

**Listing 3. Identifier-normalization pattern used to maintain a consistent wishlist state.**

JavaScript

const key = String(productId);
favorites.add(key);
const isFavorite = favorites.has(String(item.id));

**Listing 4. Recommendation endpoint (FastAPI route, simplified).**

Python

@app.get("/recommend/{user_id}")
async def get_recommendations( user_id: int, n: int = Query(default=10, ge=1, le=50), category: Optional[str] = Query(default=None), api_key: str = Depends(verify_api_key)):
 recs, _ = ml_recommender.recommend( user_id=user_id, n=n, require_diversity=True, category=category )
 return {"user_id": user_id, "count": len(recs), "recommendations": recs}

**Appendix B. Experiment Replay Protocol (Controlled Ablation)**

The following procedure reproduces the controlled ablation reported in Section 6, including the model-only, reliability-only, and combined configurations, on the fixed n = 500 session trace.

1. Create and activate a Python virtual environment, then install dependencies from the repository root: python -m venv .venv && source .venv/bin/activate && pip install -r requirements.txt
2. Ensure model and data artifacts are present, importing or generating them with the repository utilities (e.g., import_models.py and training scripts) if necessary.
3. Start backend services from the repository root, e.g., uvicorn ondc_app:app.
4. Run the baseline, model-oriented evaluation path (NCF-weighted ensemble configuration) and record ranking outputs and metrics: python evaluate_performance.py
5. Run the reliability-oriented configuration (serving-reliability fixes active, baseline ranking configuration) and collect RRSR and WCR outputs through the same trace protocol.
6. Run the combined configuration (ensemble model and reliability fixes both active) under identical request traces and the same random seed.
7. Compute and tabulate comparative metrics using the repository analysis scripts (e.g., benchmark_analysis.py) so that all three variants are compared under the same catalog snapshot.
8. For the multi-seed robustness check (Table 9, Section 6.2), repeat step 4 for LightGCN-only, GRU4Rec-only, and the NCF-based ensemble across seeds 1-46, and aggregate the per-seed MAP@10/Recall@10/NDCG@10 outputs into the mean/standard-deviation summary.
9. For the FAR breakdown (Table 10, Section 6.3), run the instrumented fault-injection harness against the post-intervention system for each of the three currently supported fault-trial types (id_type_drift, route_mismatch, schema_drift) and tabulate the handled/far/error outcome shares it reports.
10. Archive logs and generated summaries used to populate Tables 6-11 and Figs. 3-6, for traceable reproducibility.

Additional repository artifacts supporting reproducibility include: a technical report (TECHNICAL_DOCUMENTATION.md), project status and benchmark outputs (PROJECT_STATUS.md, backend benchmark outputs, and test logs in the repository root), and deployment manifests for containerized reproduction of the runtime environment.
